# In this month’s Bulletin

**DOI:** 10.2471/BLT.25.001125

**Published:** 2025-11-01

In the editorial section, Shyama Kuruvilla et al. (642) introduce this month’s special theme issue on traditional medicine and global health.

Gary Humphreys (645–646) reports on the regulatory and scientific challenges for integration of traditional medicines into health systems. Nicole Redvers speaks to Gary Humphreys (647–648) about her work at the intersection of Indigenous health, traditional medicine and planetary health, the subject of a related perspective (722–729).

## Australia, Canada, China, European Union, Republic of Korea, United States of America 

### Integration of traditional and complementary medicines

Qi Chen et al. (696–707) assess regulatory frameworks and evidence requirements.

## Barbados, Brazil, Canada, China, India, the Philippines, Qatar, Republic of Korea, the Russian Federation, South Africa, United Kingdom of Great Britain and Northern Ireland, United States of America

### National examples and global funding for research on traditional, complementary and integrative medicine

Amie Steel et al. (649–661) delineate a funding landscape.

## Region of the Americas

### Demand for traditional and complementary medicine 

Natalia Houghton et al. (730–737) explore data on use.

## United States of America

### Whole health approaches 

Alex H Krist and Tracy Gaudet (750–752) share implementation lessons.

## Global

### Hypertension, diabetes and hypercholesterolaemia 

Mubarak Ayodeji Sulola et al. (662–674) tally traditional medicine use reported in 71 surveys.

### Primary health-care systems and traditional medicines

Minmin Wang et al. (675–684) review the evidence of integration.

### Acupuncture recommendations 

Shuo Cui et al. (685–695) review the evidence used for migraine treatment guidelines.

### Innovation, ethics and intellectual property

Sumeet Goel et al. (708–714) describe requirements for balance.

### WHO’s global traditional medicine strategy 

Yuk Ming Alice Wong et al. (715–721) analyse policy implications.

### Artificial intelligence in traditional medicine

Sameer Pujari et al. (738–740) discuss policy and governance strategies.

### Triage of medicinal plants

Bhushan Patwardhan et al. (741–743) propose a global approach to safety assessment.

### International Statistical Classification of Diseases 

Ye-Seul Lee et al. (744–746) advocate for better use of diagnostic codes.

### Mitigating the side effects of cancer treatments 

Vu Thi Thu Trang et al. (747–749) call research on supportive care interventions.

